# Sir William Osler's "Give It Up"

**Published:** 1913-07-05

**Authors:** 


### The Hospital

The Workers' Newspaper of Administrative Medicine and Institutional Life, Administration, National Insurance and Health.

No. 1410, Vol. LIV. SATURDAY, JULY 5, 1913. PRICE ONE PENNY.

### SIR WILLIAM OSLER'S "GIVE IT UP."

Sir William Osler, the author of probably the best and most complete System of Medicine ever published, speaks with a freshness and authority on every subject he touches, which secures for his suggestions immediate consideration. Sir William made a model president of the Hospitals Conference at Oxford last week, and was generous enough to preside at all the sections throughout and to give close attention to the various subjects dealt with. No more successful conference of hospital managers has ever been held. Much of the success of the Oxford meeting was due to the spontaneous hospitality and goodwill extended to those present by Queen's College through Mr. G. B. Cronshaw, who made a most genial and courteous host. The occasion was full of interest from the circumstance that the Radcliffe Infirmary, Oxford, is erecting a new wing, which will give this institution the most complete clinical and pathological laboratories yet attached to a county hospital in this country. This new development, which may be traced no doubt to the influence of the Regius Professor of Medicine, backed by his colleagues, adds weight to the advice which Sir William Osler gave to county hospital managers throughout the country.

Sir William's advice is fourfold. He urges that the time has come (and in this we agree with him) when the voluntary hospital system must be extended if it is to continue as the great hospital system of the British Islands. At present these hospitals do everything for the poor, who are rich in their hospital care and treatment, but they do practically nothing for the middle classes, or the poor-rich, as Sir William defines them. The second point was that, although the general hospitals of this country are excellent so far as the care of the patient and all that appertains to its success is concerned, they lack the full equipment for the adequate treatment of disease by modern methods. In other words, every county hospital throughout the country is advised without a moment's avoidable delay to procure adequate plans and to erect and open clinical and pathological laboratories. Without these it is not possible to provide means for the best available treatment of the patient, and no efficient hospital in these days can do without such modern equipment. There should not be a single hospital throughout Great Britain which has to send to the research laboratories in London to enable a diagnosis to be made as to whether a particular case is one of carcinoma or not.

Sir William next urged, with great force and truth, that the day has gone by when the work of a county hospital can be properly done, so far as medicine is concerned, by three or four men in general practice. The developments of modern medicine make it impossible for a general practitioner to devote the necessary time for special study and research and keep himself up to date thoroughly in laboratory methods and those of research. Sir William therefore advocates the substitution of one pure physician to devote his whole time to medicine and to be in charge of the whole of the medical work at each of the county and smaller hospitals throughout the country. A hundred years ago nearly every county town in England had such a physician, and the reason for his disappearance was mainly due to the fact that there are often two or three physicians so-called where one would do. It would be a practical reform of the utmost value to the people in each community and to the general members of the profession practising within its area to maintain one physician, and he a consultant, at each hospital containing only fifty or sixty medical men. We make no doubt that as the general knowledge of modern developments in medicine spreads throughout the community, unless this reform is spontaneously effected, the public will insist upon its being carried out as the condition of their continued support of the county and smaller hospitals. The fourth recommendation is equally important to all the interests concerned in the efficiency of these hospitals. It is that every county hospital should be utilised for the purposes of graduate instruction so as to make its work of the maximum value to the practitioners residing in the district which it serves.

It is not possible to deal fully with Sir William Osler's four points in the present article. They are all admirable, and will, we have no doubt, within a few years be introduced and form part of our British system of hospitals. The one essential difficulty to be overcome is the deep-rooted conservatism which embraces the English hospital system and creates difficulties which have little real foundation apart from this conservatism. The sphere occupied by the voluntary hospital is undoubtedly too limited at present, and if it is to endure it must be extended to meet fully modern requirements. It needs the promulgation of a well thought out and practical scheme to embrace the whole voluntary hospital system, and to supply adequate hospital care for all classes of his Majesty's lieges on a paying and *pro rata* base, combined with the fullest clinical and pathological laboratories. We are confident that this reorganisation can gradually be effected with complete success. It would bring with it to the voluntary hospital system in this country new strength and popularity.

We have to evolve out of the voluntary system the best and most complete hospital organisation, with a perfected health service, that the world has ever seen. One effect of the National Insurance Act should be that this reorganisation will be carried through with the co-operation of the voluntary hospital managers and the great body of medical practitioners. Failing some such system, the Poor-Law infirmaries will be first turned into State hospitals, and with this change must gradually come, as Sir William Osler shrewdly foresees, a State service, with a rival scheme of municipal hospitals. The sooner this is appreciated by the managers of voluntary hospitals, the easier must it prove to safeguard the interests of these noble institutions. We want the hearty co-operation of all the interests involved to perpetuate these establishments by bringing them into complete harmony with modern developments in medicine. This would enable the voluntary hospitals to meet the present needs of those classes of the community which have heretofore not been able to obtain the hospital care they so urgently require.

